# One-Pot Aqueous Synthesis of Fluorescent Ag-In-Zn-S Quantum Dot/Polymer Bioconjugates for Multiplex Optical Bioimaging of Glioblastoma Cells

**DOI:** 10.1155/2017/3896107

**Published:** 2017-11-13

**Authors:** Alexandra A. P. Mansur, Herman S. Mansur, Sandhra M. Carvalho, Anderson J. Caires

**Affiliations:** ^1^Center of Nanoscience, Nanotechnology and Innovation (CeNano^2^I), Federal University of Minas Gerais (UFMG), Belo Horizonte, MG, Brazil; ^2^Department of Preventive Veterinary Medicine, Veterinary School, Federal University of Minas Gerais (UFMG), Belo Horizonte, MG, Brazil; ^3^Department of Physiology and Biophysics, ICB, Federal University of Minas Gerais (UFMG), Belo Horizonte, MG, Brazil

## Abstract

Cancer research has experienced astonishing advances recently, but cancer remains a major threat because it is one of the leading causes of death worldwide. Glioblastoma (GBM) is the most malignant brain tumor, where the early diagnosis is vital for longer survival. Thus, this study reports the synthesis of novel water-dispersible ternary AgInS_2_ (AIS) and quaternary AgInS_2_-ZnS (ZAIS) fluorescent quantum dots using carboxymethylcellulose (CMC) as ligand for multiplexed bioimaging of malignant glioma cells (U-87 MG). Firstly, AgInS_2_ core was prepared using a one-pot aqueous synthesis stabilized by CMC at room temperature and physiological pH. Then, an outer layer of ZnS was grown and thermally annealed to improve their optical properties and split the emission range, leading to core-shell alloyed nanostructures. Their physicochemical and optical properties were characterized, demonstrating that luminescent monodispersed AIS and ZAIS QDs were produced with average sizes of 2.2 nm and 4.3 nm, respectively. Moreover, the results evidenced that they were cytocompatible using* in vitro* cell viability assays towards human embryonic kidney cell line (HEK 293T) and U-87 MG cells. These AIS and ZAIS successfully behaved as fluorescent nanoprobes (red and green, resp.) allowing multiplexed bioimaging and biolabeling of costained glioma cells using confocal microscopy.

## 1. Introduction

Despite indisputable progress in medicine in the recent decades, cancer remains as one of the most devastating diseases of the 21st century challenging scientists and professionals as the word “cancer” covers approximately 200 different types of disease [1–3]. In fact, it is far more likely that advances in science for early diagnosis will result in threating more cancers as “manageable” chronic diseases with patients maintaining a relative satisfactory quality of life [4].

Brain tumors are the most common cancer occurring among children (age 0–14) and the leading cause of cancer-related deaths at that age, above leukemia statistics. Brain tumors are a diverse group of neoplasms that frequently carry a poor prognosis for patients [5, 6]. Glioblastoma (GBM, World Health Organization grade IV glioma) is the most prevalent and lethal primary intrinsic brain tumor. Unlike other solid tumor cell types, GBM widely and aggressively invades the surrounding brain but hardly metastasizes to other organs [7, 8]. Despite tremendous efforts to develop diagnostic tools and therapeutic avenues, the accurate early detection and effective treatment of brain tumors remains a difficult challenge to be overcome in the field of neurooncology. Currently, the prognosis for GBM tumor is at the extreme worst with mortality greater than 90% at 5 years, with a median survival of 12.6 months [9–11].

Modern neuroimaging tools are being applied to diagnose and grade brain tumors preoperatively, to plan and direct surgery intraoperatively, and to monitor and assess treatment response and estimating patient prognosis [12, 13]. Current research in brain tumor imaging attempts to develop, validate, and clinically implement advanced neuroimaging techniques that can benefit in the diagnosis and the detection of early treatment inefficiencies [12]. Imaging techniques including magnetic resonance imaging (MRI), computed tomography (CT), and positron-emission tomography (PET) are the most common modalities for brain tumor diagnosis, characterization, and intraoperative imaging. Other techniques such as fluorescence imaging have been developed for intraoperative fluorescence-guided tumor resection. These imaging modalities can aid in delineating the boundaries between neoplastic and normal tissue, helping doctors determine the most suitable sequence of treatment [9, 10]. Recently, theranostics, which is the combination of therapy and diagnosis, has become one of the primary keywords in cancer research, taking into account the fact that if cancer growth can be hampered during the diagnostic procedure, the subsequent cancer treatment would be facilitated because cancer growth is delayed or cancer burden is reduced [14].

To this end, nanotechnology offers a promising platform for the evolution of cancer molecular imaging strategies where nanomaterials can be used in the development of novel theranostic systems in oncology. Quantum dots (QDs) are being intensively studied as innovative class of nanoprobes for biomedical imaging because of their unique optical and electronic properties. Multiplexed molecular imaging relying on fluorescent QDs can reveal the tempospatial relationship among molecules by simultaneously staining several tumor biomarkers [6]. To overcome the obstacles of QDs for biomedical imaging, the physicochemical properties of QDs such as composition, size, shape, and surface characteristics associated with cytotoxicity have been comprehensively investigated [6, 15, 16]. However, despite the intense research in the realm of quantum dots for nanomedicine applications, the large majority of studies are based on Cd-containing semiconductor core (i.e., CdS, CdSe, and CdTe) synthesized using organometallic process at high temperature [17, 18]. For that reason, their potential toxicity has become a subject of serious discussion and debate, without a definitive conclusion yet. Some studies have demonstrated that Cd-based QDs can degrade in a biological environment, releasing highly cytotoxic Cd^2+^ ions [18, 19]. Therefore, over the past few years, a variety of Cd-free QDs have been produced from materials including zinc chalcogenides (i.e., ZnSe, ZnS), copper indium sulfide (CuInS_2_), silver indium sulfide (AgInS_2_, AIS), silver sulfide (Ag_2_S), and core-shell nanostructures (AgInS_2_-ZnS, ZAIS) [17, 19–21]. Importantly, some of these QDs share many of the advantages of commonly used Cd-based QDs regarding their optical properties and photoluminescence stability, surface biochemistry, and colloidal stability. Hence, QD-based probes that comply with the full set of requirements for biomedical applications can be used to target cancer molecules with high specificity [22–24]. Although ternary semiconductor QDs (e.g., Ag-In-S) have been already prepared in water medium for biomedical applications [19, 20, 25–28] no report was found in the consulted literature where they were directly synthesized using polysaccharide-based biopolymers as surface capping ligands for* in vitro* imaging GBM cells.

**Figure 1:**
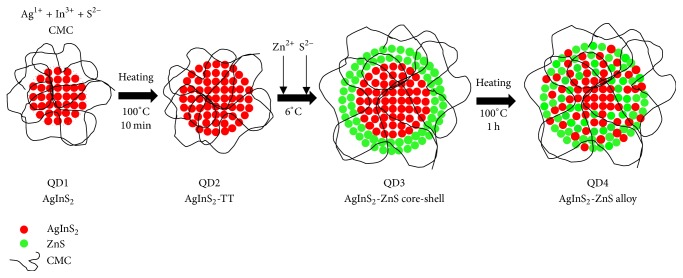
Procedure for fabrication Ag-In-S and Ag-In-Zn-S quantum dots.

Thus, in this study, a facile one-pot synthesis of novel Cd-free QDs based on AIS and ZAIS semiconductor nanocrystals produced by an eco-friendly aqueous process using carboxymethylcellulose simultaneously as stabilizing ligand and for surface biofunctionalization is presented. These colloidal nanoconjugates were cytocompatible and demonstrated fluorescent activity for effective multiplexed bioimaging of malignant glioma cells* in vitro*, as schematically depicted in Figure  1S (Graphical Abstract, Supplementary Material available online at https://doi.org/10.1155/2017/3896107). We endeavor that this research paves the way to develop new fluorescent biomarkers in nanomedicine for diagnosis, targeting and therapy of brain cancer tumors.

## 2. Materials and Methods

### 2.1. Materials

Zinc nitrate hydrate (Zn(NO_3_)_2_·6H_2_O, >99%), indium nitrate hydrate (In(NO_3_)_3_·*x*H_2_O, In = 28.5% wt), carboxymethylcellulose sodium salt (CMC, degree of substitution: 0.7; average molecular mass = 90,000 Da; medium viscosity: 180 cps, 4% in H_2_O at 25°C), 3-(4,5-dimethylthiazol-2yl) 2,5-diphenyltetrazolium bromide (MTT, >98%), Triton™ X-100, sodium dodecyl sulfate (SDS, ≥99.0%), paraformaldehyde (95%), and hydrochloric acid (HCl, 37%) were purchased from Sigma-Aldrich (USA). Silver nitrate (AgNO_3_, 99.9%) and sodium sulfide hydrate (Na_2_S·9H_2_O, >98%) were purchased from Synth (Brazil). Dulbecco's Modified Eagle Medium (DMEM), fetal bovine serum (FBS), phosphate buffered saline (PBS), penicillin G sodium, streptomycin sulfate, and amphotericin-b were supplied by Gibco BRL (USA). Hydromount was purchased from Fisher Scientific Ltd. (UK). Human embryonic kidney (HEK 293T, American Type Culture Collection, ATCC® CRL-1573™) cells were kindly provided by Professor M.F Leite (Department of Physiology and Biophysics, UFMG). Malignant glioma (U-87 MG) cells were purchased from Rio de Janeiro Cell Bank (ATCC® HTB-14™).

Aforementioned chemicals were used without further purification, deionized water (DI water, Millipore Simplicity™) with a resistivity of 18 MΩ cm was used to prepare the solutions, and the procedures were performed at room temperature (RT, 23 ± 2°C), unless specified otherwise.

### 2.2. Synthesis of Quantum Dot Conjugates

#### 2.2.1. Synthesis of AIS QDs (QD1 and QD2)

CMC solution (1% w/v) was prepared by adding sodium carboxymethylcellulose powder (0.5 g) to a 50 mL of water and stirring at room temperature until complete solubilization occurred. AgInS_2_ (QD1) conjugates were synthesized* via* an aqueous route at room temperature as follows: 2 mL of CMC solution and 48 mL of deionized water were added to a flask and the solution stabilized at physiological pH (7.5 ± 0.2). Under magnetic stirring, 0.33 mL of the silver precursor solution (AgNO_3_, 1 × 10^−3^ mol·L^−1^) and 1.33 mL of indium precursor solution (In(NO_3_)_3_·*x*H_2_O, 1 × 10^−3^ mol·L^−1^) were added to the flask and stirred for 1 min. This stoichiometry of Ag : In molar ratio of 1 : 4 gives the most intense emission for AgInS_2_ nanocrystals [20, 29, 30]. In the sequence, under vigorous stirring, 2.0 mL of sulfur solution precursor (Na_2_S·9H_2_O, 1 × 10^−3^ mol·L^−1^) was dropped into the flask and stirred for 10 min.

In order to improve the optical properties of AgInS_2_ cores (referred to as QD1 or AgInS_2_), some procedures were taken in following sequence: (a) thermal treatment (TT) of AgInS_2_ cores at 100 ± 5°C for 10 min to annealing/growth of AgInS_2_ QDs (referred to as QD2 or AgInS_2_-TT); (b) ZnS shell growth overlaying AgInS_2_-TT cores (referred to as QD3 or AgInS_2_-ZnS core-shell); and (c) thermal treatment of AgInS_2_-ZnS core-shell structure at 100 ± 5°C for 60 min leading to alloying (referred to as QD4 or AgInS_2_-ZnS alloy).  Figure 1 summarizes the steps for the preparation of ZAIS QDs from AIS QDs that are described in Section 2.2.2.

#### 2.2.2. Synthesis of ZAIS QDs (AgInS_2_-ZnS, QD3, and QD4)

In this step, initially an “adlayer” of ZnS was grown onto AgInS_2_-TT core reaching a core-shell nanostructure (AgInS_2_-ZnS core-shell or QD3). Therefore, the previous AIS solution (QD2) was refrigerated at 6 ± 2°C for at least 6 h. Then, at 6 ± 2°C and under stirring, a 1.25 mL of zinc precursor (Zn(NO_3_)_2_·6H_2_O, 1 × 10^−3^ mol·L^−1^) was added dropwise (10 *μ*L at each 10 s) into 50 mL of QD2 suspension followed by addition of 1.25 mL of sulfur precursor (Na_2_S·9H_2_O, 1 × 10^−3^ mol·L^−1^) in the same way (dropwise, 10 *μ*L at each 10 s) and stirred for 20 min. The resulting suspension (QD3, AgInS_2_-ZnS) was reserved for 24 h at 6 ± 2°C.

In the sequence, the QD3 suspension was heated for 1 h at 100 ± 5°C to allow interdiffusion of Zn^2+^ ions from shell into the core and annealing, leading to the formation of AgInS_2_-ZnS alloys (QD4).

All of the QDs colloidal dispersions produced were stable, homogeneous, and light brown. The QD colloids were dialyzed for 24 h (with water changes after 2 h and 4 h) against 3 L of DI water using a Pur-A-Lyzer™ Mega Dialysis Kit (Sigma, cellulose membrane with molecular weight cut-off filter, MWCO of 12,000 Da) under moderate stirring at room temperature. After purification, the QD dispersions were stored at RT until further use. Colloidal dispersions were concentrated using an Amicon® Ultra Filter (Millipore) with a 100,000 molecular mass (*M*_*W*_) cut-off cellulose membrane.

### 2.3. Characterization of Quantum Dot Conjugates

Ultraviolet-visible (UV-vis) spectroscopy measurements were performed using Perkin-Elmer, Inc. (USA) equipment (Lambda EZ-210) in transmission mode with samples in a quartz cuvette over a wavelength range between 600 and 190 nm. All of the experiments were conducted in triplicate (*n* = 3) unless specifically noted, and the data were presented as the mean ± standard deviation.

The photoluminescence spectroscopy (PL) of the conjugates was performed based on spectra acquired at RT using a violet diode laser module at 405 nm excitation wavelength (*λ*_exc_) (150 mW, Roithner LaserTechnik, Germany) coupled to a USB4000 VIS-NIR (visible-near infrared) spectrophotometer (Ocean Optics, Inc., USA). All of the tests were performed using a minimum of four repetitions (*n* ≥ 4). Quantum yield (QY) was measured according to the procedure using Rhodamine 6G (Sigma, USA) in ethanol as the standard at *λ*_exc_ = 405 nm [31]. Additionally, QD colloidal media were placed inside a “darkroom-chamber” where they were illuminated by a UV (ultraviolet) radiation emission bulb (*λ*_exc_ = 365 nm, 6 W, Boitton Instruments). Digital color images were collected when the QDs fluoresced in the visible range of the spectra.

Morphological characterization of QD nanostructures was based on the images, electron diffraction patterns (ED), and energy-dispersive X-ray spectra (EDX) using Tecnai G2-20-FEI (FEI Company, USA) transmission electron microscope (TEM) at an accelerating voltage of 200 kV. In all of the TEM analyses, the samples were prepared by placing a drop of a dilute QD suspension onto carbon-coated copper grids (Electron Microscopy Sciences, USA) and allowing them to dry at room temperature overnight. The QD size and size-distribution data were obtained based on the TEM images by measuring at least 150 randomly selected nanoparticles using image processing program (ImageJ, version 1.50, public domain, National Institutes of Health) [32].

X-ray diffraction (XRD) patterns were recorded using PANalytical (UK) Empyrean diffractometer (Cu-K*α* radiation with *λ* = 1.5406 Å). Measurements were performed in the 2*θ* range from 6 to 60° with steps of 0.017°. For the sample preparation, concentrated colloidal QD dispersions were placed onto glass slides and oven-dried at 40 ± 1°C for 12 h.

Dynamic light scattering (DLS) and zeta potential (ZP) analyses were performed using ZetaPlus instrument (Brookhaven Instruments Corporation, 35 mW red diode laser light, wavelength *λ* = 660 nm) with a minimum of ten replicates. The ZP measurements were performed at 25 ± 2°C under the Smoluchowski approximation method with a minimum of ten replicates.

### 2.4. Biological Characterization of QD Conjugates

All of the biological tests were conducted according to ISO 10993-5:2009/(R)2014 (Biological evaluation of medical devices: tests for* in vitro* cytotoxicity) using kidney cell line of a human embryonic culture (HEK 293T) and malignant glioma cells (U-87 MG). HEK 293T (passage 18) and U-87 MG (passage 8) cells were cultured in DMEM with 10% FBS, penicillin G sodium (10 units mL^−1^), streptomycin sulfate (10 mg mL^−1^), and amphotericin-b (0.025 mg mL^−1^) in a humidified atmosphere of 5% CO_2_ at 37°C.

#### 2.4.1. Evaluation of Cytotoxicity by MTT Cell Viability Assay

MTT (3-(4,5-dimethylthiazol-2yl) 2,5-diphenyl tetrazolium bromide) experiments were performed to evaluate the toxicity of QDs dispersions. HEK 293T and U-87 MG cells were plated (1 × 10^4^ cells/well) in 96-well plates. Cell populations were synchronized in serum-free media for 24 h. After that, the media volume was suctioned and replaced with DMEM media containing 10% FBS for 24 h. The samples of QD1, QD2, QD3, and QD4 colloidal solutions were added to individual wells at final concentrations of 2.5 nmol·L^−1^ of QD nanoparticles (~1 mg mL^−1^). For MTT assay, control samples were designed as follows: control group (cell culture with DMEM medium); positive control (1.0% v/v Triton™ X-100 in PBS); and negative control (chips of sterile polypropylene Eppendorf, 1 mg mL^−1^, Eppendorf, Germany). After 24 h, all media were aspirated and replaced with 60 *μ*L of culture media containing serum to each well and images of cells were acquired on an Leica DMIL LED (Germany) inverted microscope. Then 50 *μ*L of MTT (5 mg mL^−1^) was added to each well and incubated for 4 h in an oven at 37°C and 5% CO_2_. Next, 40 *μ*L SDS solution/4% HCl was placed in each well and incubated for 16 h in an oven at 37°C and 5% CO_2_. Then, 100 *μ*L was removed from each well and transferred to a 96-well plate. The absorbance was measured at 595 nm on iMark™ Microplate Absorbance Reader (Bio-Rad) with a 595 nm filter. Percentage cell viability was calculated according to (1). The values of the controls (wells with cells and no samples) were set to 100% cell viability.

(1) Cell  viability=Absorbance  of  sample  and  cellsAbsorbance  of  control×100%.

#### 2.4.2. Cellular Uptake of QD Conjugates by Laser Scanning Confocal Microscopy: Internalization, Kinetics, and Multiplexed Images

The evaluation of the QDs conjugates as fluorescent biological probes was performed using confocal laser scanning microscopy after exposing HEK 293T and U-87 MG cells to QD1 and QD4 samples. QD1 (AIS) with the lowest QY was chosen for cell imaging with two cell lines and cellular uptake kinetics to demonstrate the feasibility of using these QD conjugates for cell imaging due their unique optical emission properties. QD1 (AIS) and QD4 (ZAIS) were selected as the fluorescent species for spectrally multiplexed imaging of cells considering the detectable wavelength of PL emission. HEK 293T cells on passage 19 and U-87 MG cells on passage 9 were plated (5 × 10^5^ cells per well) in 6-well plate. The cells were incubated for 4 days in 5% CO_2_ at 37°C and synchronized for 24 h. Then, QD colloidal suspensions with the medium solution (DMEM with 10% FBS) were added to the cells and incubated in 5% CO_2_ at 37°C from 30 min up to 120 min (in cellular uptake kinetics study), followed by washing with PBS. For evaluation of internalization and kinetics, QD1 was incubated at final concentration of 50 nmol·L^−1^ of QD nanoparticles. For costained multiplexed imaging, QD1 and QD4 were incubated separately at final concentration of 50 nmol·L^−1^ or QD1 and QD4 were incubated together, at the same concentration of each. In the sequence, the cells were fixed with paraformaldehyde (4%) for 30 min and washed three times with PBS, and cover slips were mounted with Hydromount. Images were obtained with a Zeiss LSM Meta 510 confocal microscope (Carl Zeiss, Germany) using the water immersion (objective 63x Plan-Apo/1.4 NA, Numerical Aperture). For green-emission, argon laser was used to excite at *λ*_exc_ = 488 nm and emission was collected at 505–550 nm. For red-emitting QD, excitation was at *λ*_exc_ = 568 nm and emission at LP 585 nm (LP = low pass). For the reference control, cells were incubated only with the original medium with 10% FBS (autofluorescence). Plot of intensity profiles and measurements of mean fluorescence intensity were performed using public domain image processing software (ImageJ software, version 1.50).

## 3. Results and Discussion

### 3.1. Characterization of Quantum Dot Conjugates

**Figure 2:**
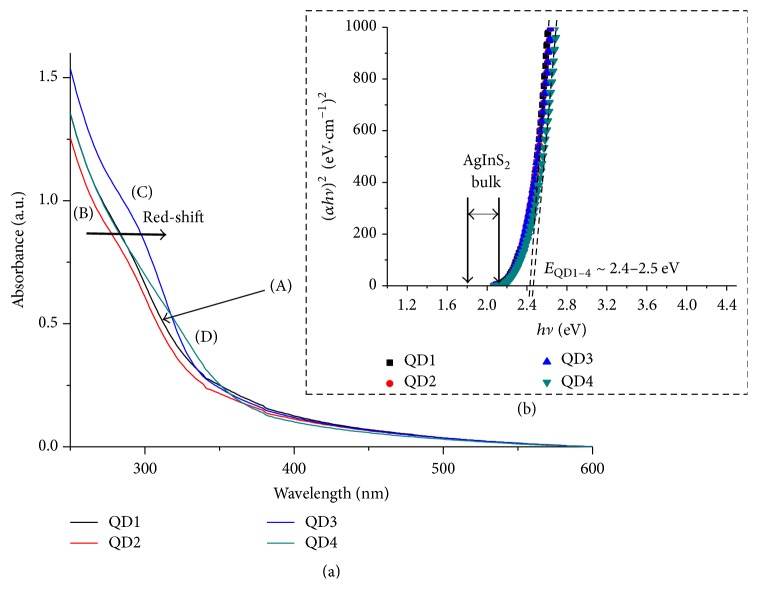
UV-vis absorption spectra (a) and optical absorption spectra (Tauc relation) (b) for QD1 (A), QD2 (B), QD3 (C), and QD4 (D).

Here, carboxymethylcellulose was used as the ligand for stabilizing the ultra-small semiconductor nanocrystals in water media, which were characterized in situ by UV-vis spectroscopy. These QDs presented broad UV-vis absorption spectra with an onset at approximately 650 nm (Figure 2(a)). This relatively broad absorption edge has been reported in literature [27, 33, 34] for AIS and ZAIS nanoparticles, which was generally associated with size distribution of QD produced [35, 36]. The arrow in Figure 2(a) shows the “red-shift” in the absorption spectra of QD2 after overcoating with the ZnS passivating shell (QD3). Such behavior is caused by the growth of the wider bandgap (ZnS, bulk band gap (*E*_*g*_) = 3.61 eV [37]) semiconductor, which may be interpreted as a partial leakage of the exciton from the AgInS_2_-core (h^+^/e^−^, holes/electrons) into the ZnS shell through the heterojunction [38].

Band gap values for the prepared QDs were extracted from the UV-vis absorbance curves using the “Tauc relation” (Figure 2(b)). AgInS_2_ (AIS) is a direct bandgap semiconductor with *E*_*g*_ ranging from 1.8 eV (tetragonal) to 2.1 eV (orthorhombic) [27, 39]. The estimated *E*_QD_ (QD band gap) values were between 2.4 eV and 2.5 eV, which are larger than that of the bulk values (*E*_*g*_ = 1.8–2.1 eV) due to the quantum confinement effect.

**Figure 3:**
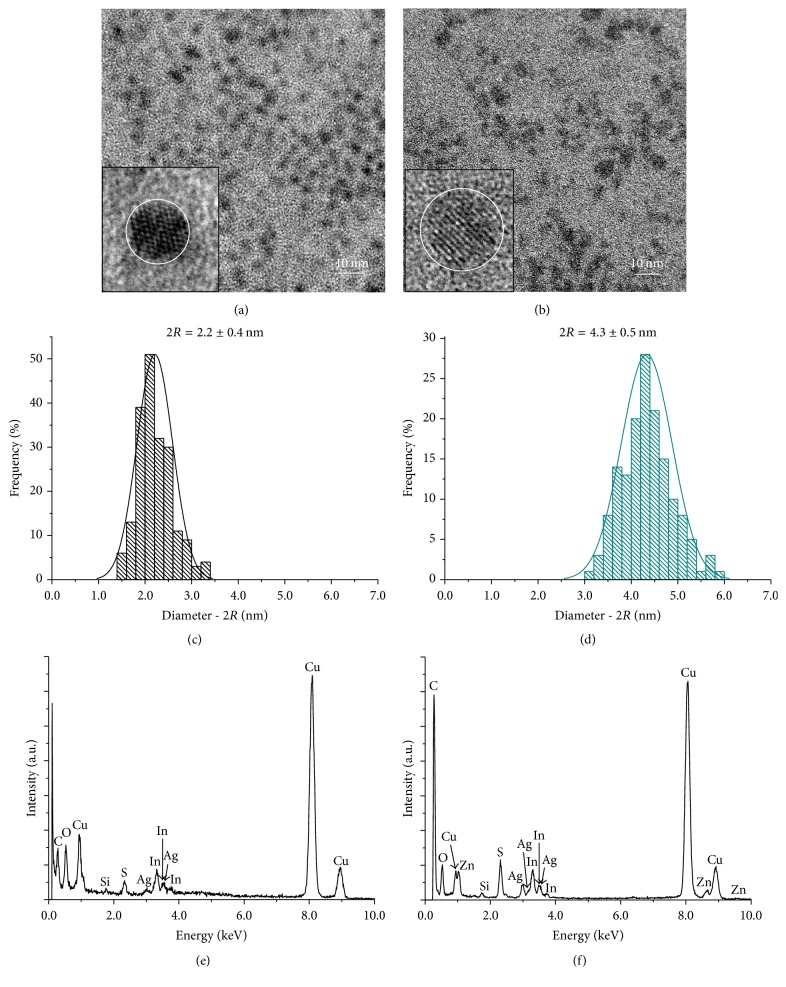
TEM image (inset: HRTEM picture) for QD1, AIS core (a) and QD4, ZAIS (b), histograms of size distribution for QD1 (c) and QD4 (d), and EDX spectra for QD1 (e) and QD4 (f).

In order to access the morphological features, the sizes, and the elemental composition of the QDs, TEM analysis coupled with EDX was performed. AgInS_2_ core (AIS, QD1, Figure 3(a)) and quaternary alloyed AgInS_2_-ZnS (ZAIS, QD4, Figure 3(b)) typical images revealed the formation of relatively monodispersed nanoparticles with reasonable spherical shape. The clear continuous lattice fringes obtained by electron diffraction in the HRTEM (high-resolution transmission electron microscopy) images demonstrated the single-crystalline nature of the QDs. The histograms of nanoparticle size distributions (Figures 3(c) and 3(d)) indicated the average size of 2.2 ± 0.4 nm and 4.3 ± 0.5 nm for QD1 and QD4, respectively. The observed increasing in diameter was expected by considering the growth of the ZnS shell over the nanocrystal core. EDX measurements confirmed the presence of Ag, In, and S in ternary QD1 and the incorporation of Zn in the alloyed QD4 (Figures 3(e) and 3(f)). For QD1, EDX analysis indicated an average molar ratio of [Ag] : [In] = 1 : 4.3, which is in good agreement with the precursor molar ratio for metal cations [1 : 4].

**Figure 4:**
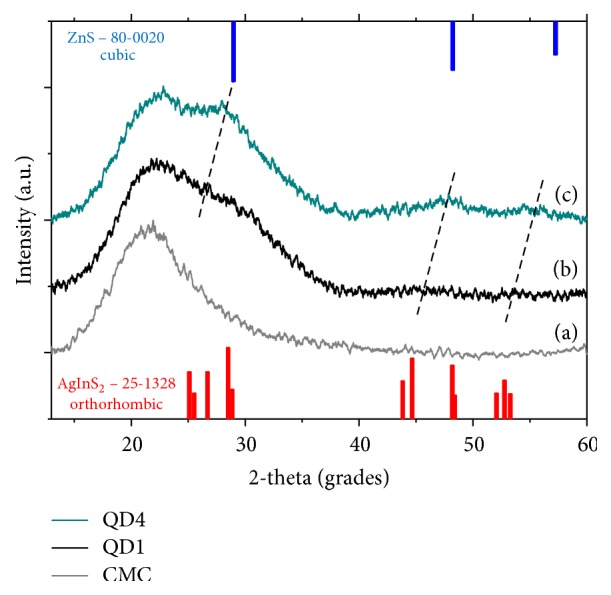
XRD patterns of CMC ligand (a), QD1 (AIS) (b), and QD4 (ZAIS) (c).

The XRD pattern indicated three broadened and weak reflections in ternary core (QD1, Figure 4(b)) and quaternary alloyed (QD4, Figure 4(c)) samples, due to the small particle size, overlapped with the broad band present at 2*θ* ~ 21.8° (Figure 4(a)) that is characteristic of the CMC polymer used for the chemical stabilization of the QDs [40]. Based on reflection positions, the orthorhombic crystal phase was suggested for AgInS_2_ nanoparticles. However, as reported in literature [27, 41], the occurrence of other AIS phases cannot be either ruled out or confirmed due to the large width of the reflections. It is clear that the diffraction peaks of ZAIS nanocrystals (QD4) were shifted to higher angles in comparison to AgInS_2_ core (QD1) due to ZnS alloying with the reflections located between the corresponding peaks of the bulk orthorhombic AgInS_2_ (International Centre for Diffraction Data, ICDD 25-1328) and cubic ZnS (ICDD 80-0020). Therefore, this feature confirmed that the QD4 (Figure 4(c)) formed an alloyed solid solution and not a mixture of AgInS_2_ and ZnS nanoparticles [21, 42].

**Figure 5:**
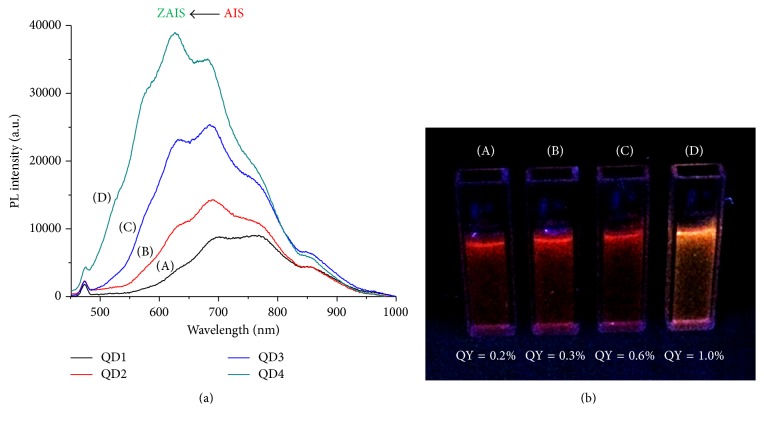
Photoluminescence spectra (a) and digital image of QD colloidal solutions excited by ultraviolet light (*λ*_exc_ = 365 nm) and QY values (b) obtained from QD1 (A), QD2 (B), QD3 (C), and QD4 (D) nanoconjugates.

Typical room temperature PL spectra of the quantum dots and QY results of QD1 to QD4 are presented in Figure 5. The spectra (Figure 5(a)) indicated that luminescence is based on defect-activated sites, where no excitonic emission was observed. In addition, “Full Width at Half Maximum” (FWHM) was larger than 100 nm, which is consistent with previous reports for Ag-In-S- and Zn-Ag-In-S semiconductor nanocrystals [43, 44].

According to the literature [29, 41] emissions of I-III-VI nanocrystals are dominated by radiative recombination related to intrinsic donor-acceptor defects due to the failure of aligned orientation between Ag and In, in addition to nonradiative pathways from surface defects due to the high surface to volume ratio of QDs. Thermal treatment (QD1 → QD2) and shell growth (QD2 → QD3) reduced the surface trap states that cause the nonradiative pathways increasing the radiative emissions, as could be clearly seen by the increase of the PL intensity (Figure 5(a)) and QY values (Figure 5(b)). Besides passivating dangling bonds on QD surface, ZnS adlayer also promoted a blue-shift of the emission spectrum. Finally, thermal treatment of core-shell structure (QD3 → QD4) greatly improved photoluminescence intensity associated with interdiffusion of Zn ions into the AIS lattice upon alloying process and further annealing [20]. In this sense, the proposed strategy of improving optical properties of AgInS_2_ by a sequence of steps was effective. Despite the relative low values of QY (*ca.* 0.2%–1.0%), which is commonly observed for QD produced in water medium at room temperature mostly due to the density of crystalline and surface defects, the drastic increase of approximately 200% and 400% after the formation of the core-shell nanostructure and after the alloying-annealing process, respectively, is remarkable.

Thus, it is important to highlight that, compared with previous reported studies [20, 27, 28], mostly based on heating up, hot injection, and organic processes, these AgInS_2_ QDs were produced using a facile one-step synthesis in aqueous media based on carboxymethylcellulose as polymer stabilizer, which offer several advantages: (a) more reproducible; (b) low-cost; (c) environment friendly; (d) commercially availability and abundance; (e) biocompatibility for further biomedical applications. However, a facile and “green” mild process using aqueous medium for the preparation of AgInS_2_/polymer nanoconjugates with high luminescent properties (i.e., PL quantum yield, QY > 40%) for augmenting bioimaging sensitivity is beyond the scope of the current study and remains a challenge for the future researches. Nonetheless, this is not a restriction, and QDs produced by aqueous processes with lower values of QY (i.e., typically < 5%) have been successfully applied for numerous applications in bioimaging (e.g., confocal microscopy, flow cytometry) [16, 24, 45].

For biological applications, it widely known that size, shape, composition, and surface chemistry of nanoparticles have important roles in the biological responses of cells, tissues, and organs. Nanoparticle surface charge was determined by zeta potential (*ξ*) measurements. The *ξ*-values for the synthesized nanoconjugates were between −43 mV and −46 mV, which indicated the predominance of negatively charged surface due to the carboxylic groups (R-COO^−^) of anionic CMC ligand at physiological pH (p*k*_*a*_ ~ 4.3) [46, 47]. In addition, the zeta potential (*ξ*-values) values lower than −40 mV indicated that the nanoparticles were electrostatically stabilized by the cellulose-modified polymer ligand as a colloidal nanoconjugate. The DLS technique was used to evaluate hydrodynamic sizes of the colloidal QDs in the medium. After the synthesis, in water medium at physiological pH, the sum of contribution of QD inorganic core with the CMC organic shell and its interactions with the surrounding medium resulted in a hydrodynamic diameter ranging from 38 nm to 48 nm. These results indicated that colloidal QD nanoconjugate suspensions contain individual nanoparticles electrostatically stabilized with negative surface charge.

In order to investigate the possible changes of surface charges and hydrodynamic sizes of QD1 and QD4 nanoconjugates immersed in the biological media for MTT and cellular uptake assays, ZP and DLS measurements were performed after 30 min of incubation (DMEM with 10% FBS). The DMEM contains inorganic salts, amino acids, vitamins, and D-glucose and is usually supplemented with FBS, which is a complex mixture (i.e., growth factors, proteins, vitamins, trace elements, and hormones) important for the growth and maintenance of cells [48–50]. After incubation with DMEM (with 10% FBS), the average zeta potential measured for both systems decreased from* ca.* −45.0 mV to −5.0 mV and the hydrodynamic radius was reduced from* ca.* 40 nm to 20 nm. These changes are associated with the overall balance of the neutralization of CMC polymer surface charges by inorganic salts and interaction of QD polymeric shell with the biomolecules from DMEM and FBS. Thus, QDs in the medium of biological assays are not agglomerated and coated with an hybrid shell (CMC-biomolecules-ions) that stabilize the near neutral QD surfaces by steric hindrance at sizes of approximately 20 nm [51, 52].

### 3.2. Biological Characterization of QD Conjugates

#### 3.2.1. Evaluation of Cytotoxicity by MTT Cell Viability Assay

The cytotoxicity of the heavy-metal free AIS (QD1 and QD2) and ZAIS (QD3 and QD4) nanoconjugates was assessed using the enzyme-based MTT. According to the study recently published [18], MTT assay has been the predominant assay for* in vitro* evaluation of nanomaterials for biomedical applications [53–55]. Two cell lines, HEK 293T and U-87 MG, were used in the experiments. HEK 293T is a permanent cell line established from primary embryonic human kidney that is widely used as cell model and is very useful for transfection experiments, as they have a higher transfection efficiency than other cell lines, making it a common choice of cultures for biological research. The U-87 MG cell line is human primary glioblastoma cell line that is commonly used for brain cancer research [56]. The choice of use malignant glioma cells is due to the high lethality of brain tumors and the limited treatment options currently available [57] that demands studies of novel nanomaterials for targeting, detection, and treatment of brain tumors at the same time, which will be helpful to the earliest diagnosis and prolong survivability for patients.

**Figure 6:**
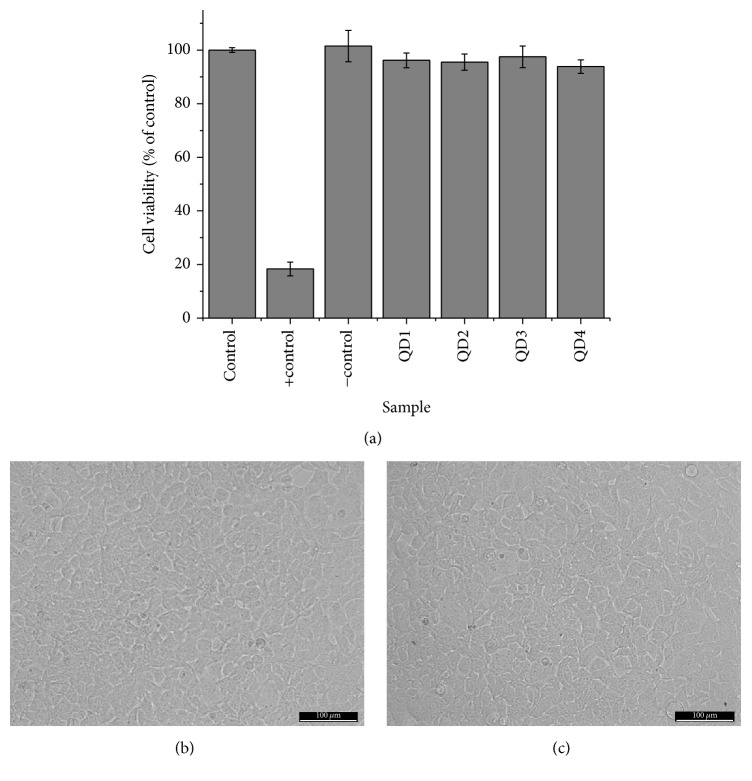
HEK 293T cell viability response by MTT assay after 24 h of incubation in direct contact with the QD nanoconjugate samples (a). HEK 293T cells images in control (b) and QD1 sample (c) (scale bar = 100 *μ*m).

**Figure 7:**
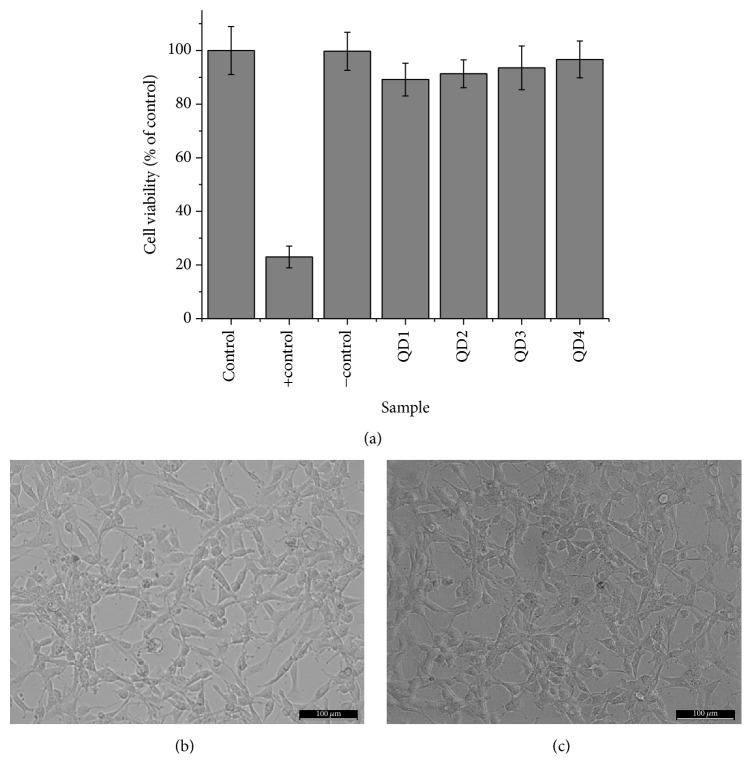
U-87 MG cell viability response by MTT assay after 24 h of incubation in direct contact with the QD nanoconjugate samples (a). U-87 MG cells images in control (b) and QD1 sample (c) (scale bar = 100 *μ*m).

The results of HEK 293T (Figure 6(a)) and U-87 MG (Figure 7(a)) cell lines in contact with the quantum dot-CMC nanoconjugates demonstrated that no significant differences in the cell viability compared to the control were detected (within the statistical variation). All of the samples presented cell viability responses typically above 90%, indicating the nontoxicity of these bioconjugates. Even HEK 293T cells, which are more susceptible to be affected by the physicochemical characteristics of the nanoparticles due to the permeability of their membranes [58], presented cell viability higher than 94% at the concentration of nanoconjugates of 1.0 mg mL^−1^ after incubation for 24 h. Optical images of the cells before (Figure 6(b)) and after incubation with QD1 nanoconjugates are in agreement with MTT results presenting more than 90% of cell confluence for HEK 293T cells (Figure 6(c)) and above 80% for U-87 MG cells before (Figure 7(b)) after (Figure 7(c)) incubation. Similar results of cell confluence were obtained for the other nanoconjugate samples in contact with HEK 293T and U-87 MG cells (Figures  2S and 3S, Supplementary Material). In addition, qualitative morphological evaluation of the cells after incubation for 24 h with QDs was performed in zoomed-in images of the cells (Figures  4S and 5S, Supplementary Material) according to the scoring system described in ISO 10933-5, where grade 0 corresponds to none reactivity and grade 4 corresponds to severe reactivity. The changes observed for all nanoconjugates and cell types under evaluation can be graded as 1 (Slight), which means* “not more than 20% of the cells are round, loosely attached and without intracytoplasmatic granules, or show changes in morphology; occasional lysed cells are present; only slight growth inhibition observable.”* The evaluation of grade 1 of our samples is considered a noncytotoxic effect according to ISO 10933-5. Thus, these nanoconjugates designed and produced with Cd-free inorganic core (AIS and ZAIS) and directly stabilized with carboxymethylcellulose ligand* via* aqueous route at physiological pH hold promise for biomedical bioimaging and targeting of cancer cells. However, the* in vitro* MTT assay was specifically used to evaluate the mitochondrial function and cell viability as a preliminary quantitative assessment of the cytocompatibility towards these nanoconjugates and further studies are required before* in vivo* or clinical applications.

#### 3.2.2. Cellular Uptake of QD Conjugates by Laser Scanning Confocal Microscopy

*(1) Cell Imaging and Kinetics of Cellular Uptake*. In this study, in order to demonstrate the unique optical properties of quantum dots as compared to conventional dyes, AIS nanoparticles with low QY (QD1, AgInS_2_, QY = 0.2%) were used as biological biomarkers and for cellular uptake evaluation using confocal laser scanning microscopy performed after incubation with HEK 293T and U-87 MG cells.

**Figure 8:**
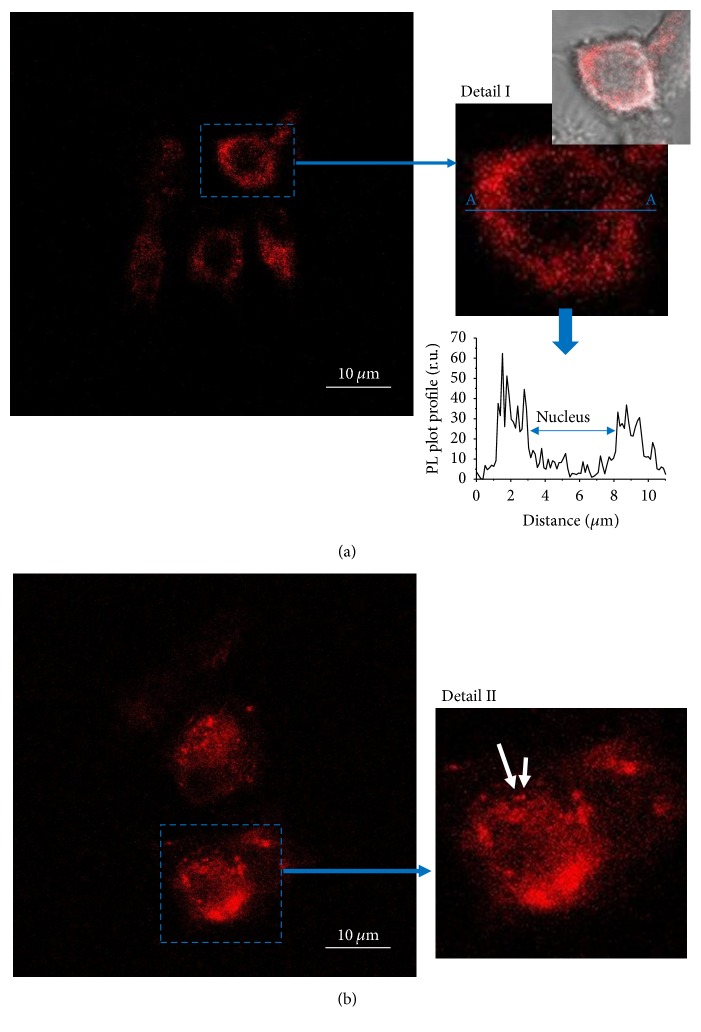
Confocal microscopy imaging of the cellular uptake of the AIS (QD1) nanoconjugates in the U-87 MG (a) and HEK 293T (b) cells. Detail I: intensity fluorescence profile along A-A line U-87 MG cell line indicating cellular localization of AIS nanoconjugates. Detail II: white arrows pointing out high fluorescent areas that may suggest the presence of vesicles filled with QDs.

Distinct from AIS/ZAIS QDs reported in literature that required biological targeting vector and high quantum yield for allowing cellular imaging [20, 44], the novel AgInS_2_ nanoconjugates developed in this study were effectively internalized by HEK 293T and U-87 MG cells, which showed clear PL red-emission after 30 min of incubation (Figure 8). For both cell lines, PL emission associated with cellular localization of AIS nanoconjugates was examined using intensity fluorescence profiles obtained using image process software (ImageJ, v1.50). Thirty minutes after cellular uptake of HEK 293T and U-87 MG cells, the distribution of AIS conjugates fluorescence emission was observed at cytosol with no obvious fluorescence in their nuclei (Detail I, Figure 8(a)) and no clear evidence of specific intracellular localization. In addition, despite the scattered red fluorescence in the cytoplasmic matrix, some high fluorescent areas were observed in the cytoplasm that may suggest the presence of vesicles filled with QDs (white arrows, Detail II, Figure 8(b)). However, in order to prove this hypothesis, costaining vesicles components is necessary.

**Figure 9:**
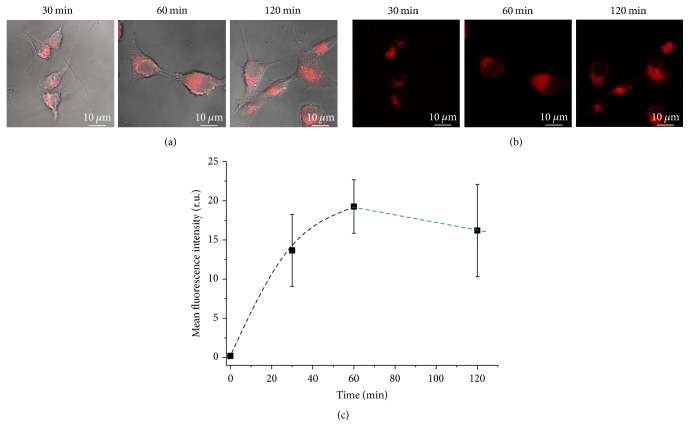
Confocal microscopy imaging of the cellular uptake of the AIS (QD1) nanoconjugates by U-87 MG cells* versus* incubation time (30 min, 60 min, and 120 min): PL + bright field image (a), PL image (b), and plot of MFI × time (c).

Also, the kinetics of cellular uptake of QD1 in glioblastoma cells (U-87 MG) were evaluated (Figure 9). Confocal fluorescent images (Figure 9(b)) were taken after 30 min, 60 min, and 120 min of incubation with the nanoconjugates and mean fluorescence intensity (MFI, Figure 9(c)) was calculated using image processing software (public domain, ImageJ, v.1.5). A significant enhancement of the fluorescence intensities associated with the increase of incubation time from 30 min to 60 min (two-sample *t*-test, one tailed, with significance level (*α*) 0.025 at 29 degrees of freedom) was detected. It is demonstrated that these nanoparticulate systems not only effectively penetrated through cell membranes allowing biolabeling but also proved the continuing endocytosis by the cells with further intracellular scattered distribution within the cytoplasmic matrix after 60 min of contact of QD with cells. At 120 min of incubation, it was observed that the mean fluorescence intensity was not significantly distinct from the obtained at 60 min of incubation (two-sample *t*-test, one tailed, with significance level (*α*) 0.025 at 29 degrees of freedom), suggesting that the saturation stage of intracellular uptake was reached [59]. In addition, for all evaluated incubation times, the cells demonstrate normal and clear morphology (Figure 9(a)).

**Figure 10:**
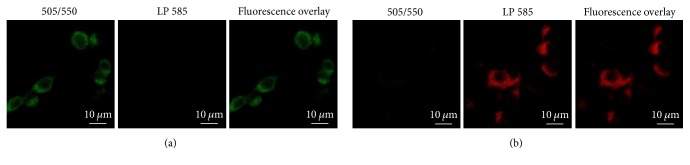
Fluorescence spectral imaging of U-87 MG cells separately treated for 30 min with green-emitting ZAIS (a) and red-emitting AIS (b) with PL emissions split by optical filters (green: 505/550 and red: LP 585) and overlaid PL image.

**Figure 11:**
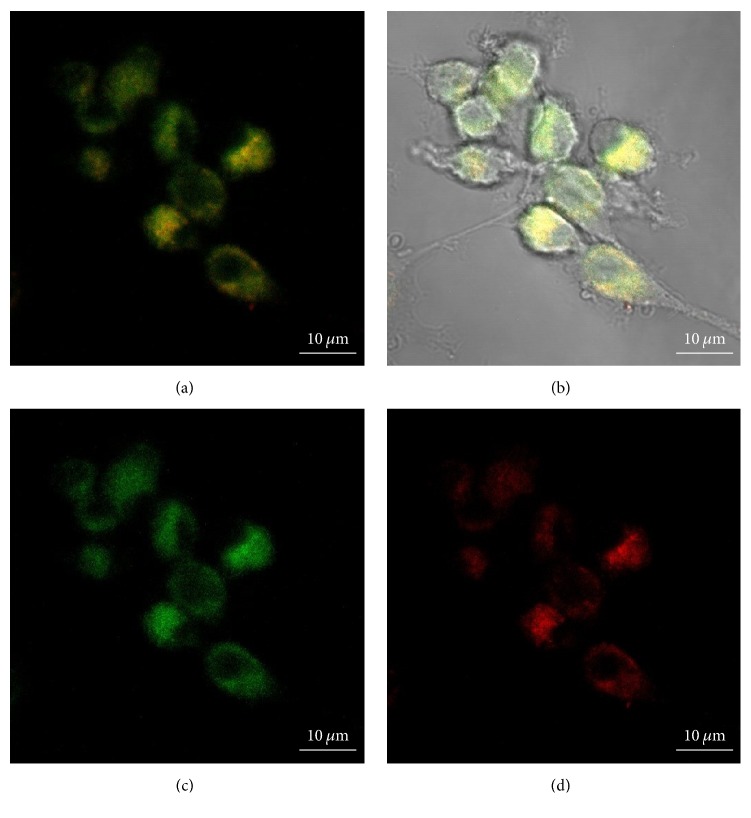
Multiplexed QD images of U-87 MG cells costained with AIS (QD1) + ZAIS (QD4): fluorescence overlay multicolor image (a); PL multicolor image overlaid with bright field image (b); PL image from filter 505/550 (green-emitting ZAIS) (c); and PL image from filter LP 585 (red-emitting AIS) (d).

*(2) Multiplexed Bioimaging*. The unique optical and electronic properties of quantum dots, such as high brightness, high chemical and photostability, continuous absorption, and relative narrow emission bandwidth, make them ideal choice as labels to develop fluorescent-based characterization techniques for detection and imaging cancer cells and tissues. In addition, QDs can be synthesized with distinct emission colors (i.e., multicolor nanoprobes* via* chemical composition and sizes) conjugated with functional biomolecules (e.g., antibodies and peptides) providing multiplexing capabilities to simultaneously identifying multiple biological targets of cancer cells and tissue for a myriad of applications in oncology [9, 10, 60–64]. In order to investigate the potential of the AIS (QD1) and the ZAIS (QD4) quantum dots for spectrally multiplexed imaging, the U-87 MG glioblastoma cells treated separately with these QDs were imaged by confocal microscopy and the fluorescent signals from the red-emitting (AIS) and the green-emitting (ZAIS) were resolved by using spectrally matched filters (505/550 and LP 585). Based on the cell images depicted in Figure 10, the results demonstrated that the potential of using the 2-dimensional encoded QDs for spectrally multiplexed imaging and the images of cell costained with AIS + ZAIS (QD1 + QD4) is shown in Figure 11 that demonstrated the feasibility of combining these QDs for multiplexed bioimaging.

It is important to highlight that these results of multiplexed imaging by confocal microscopy relied on the exceptional optical properties of the water-soluble and cytocompatible QD nanoconjugates (i.e., red-emitting, AIS, and green-emitting, ZAIS). This strategy can be transferred to other characterization techniques for cancer biomarker detection such as flow cytometry (FC), immunohistochemistry (IHC), immunohistofluorescence (IHF), image guided surgery, and steady state fluorescence [9, 10, 61–65]. Certainly, further studies are required to exploit the myriad of possibilities for producing cadmium-free QD nanoconjugates using aqueous processes with tunable PL properties (ranging from infrared to ultraviolet emission)* via* nanocrystal size and chemical composition, alloying and doping, core-shell nanostructures, processing routes, capping ligands, and others. To that end, a scenario could be envisioned for the future in which the use of multicolor QD-based fluorescent nanoconjugates could enhance the sensitivity, specificity, and multiplexing capabilities of molecular histopathology and early diagnosis of cancer for* in vitro, in vivo*, ex vivo applications. In particular, patients with glioblastoma (GBM), an extremely aggressive clinical phenotype of brain cancer, will undoubtedly benefit from the development of a new generation of diagnosis and therapies based on QD nanoconjugates with multiplexing technologies.

## 4. Conclusions

In this work a facile and eco-friendly method for synthesizing novel ternary AgInS_2_ (AIS) and quaternary AgInS_2_-ZnS (ZAIS) fluorescent colloidal nanocrystals with carboxymethylcellulose (CMC) as capping ligand using a one-pot aqueous processing route at room temperature and physiological pH was developed. The formation of colloidal semiconductor solution was monitored in situ by UV-vis spectroscopy, where the initial blue-shift of spectrum indicated the production of stable AIS and the posterior red-shift evidenced the growth of a ZnS layer resulting on the ZAIS core-shell nanostructures. TEM results indicated that monodispersed AIS and ZAIS QDs were produced with average sizes of 2.2 nm and 4.3 nm, respectively. In addition, these nanoconjugates showed surface charge determined by zeta potential measurements typically ranging from −43 mV to −46 mV and hydrodynamic diameter from 38 nm up to 48 nm assessed by the DLS method. These results indicated that colloidal QD nanoconjugates were electrostatically stabilized by negatively charged CMC polymer ligand (i.e., carboxylic groups, R-COO^−^). Moreover, the MTT results evidenced that they were preliminarily cytocompatible using* in vitro* assay with HEK 293T and U-87 MG cells. Moreover, these novel AIS and ZAIS QDs surface modified by CMC showed appropriate intracellular photoluminescence upon incubation with HEK 293T and U-87 MG cells, providing the effective function of cell bioimaging. Finally, these AIS and ZAIS QDs demonstrated red and green photoluminescent emissions, respectively, allowing effectively multiplexed bioimaging and biolabeling of costained glioma cells using confocal microscopy. Therefore, these bioconjugates offer promising nanoplatforms for potential* in vitro* and* in vivo* biomedical applications in multimodal bioimaging and targeting of cancer cells, opening a vast realm of possibilities to be explored in future researches.

## Supplementary Material

Figure 1S: Schematic representation of fluorescent nanoconjugates based on AIS and ZAIS quantum dots with CMC ligands for multiplexed bioimaging of malignant glioma cells.
Figure 2S: HEK 293T cells images after 24 h of incubation with QD2 (a), QD3 (b), and QD4 (c) nanoconjugate samples (Scale bar = 100 µm).
Figure 3S: U87-MG cells images after 24 h of incubation with QD2 (a), QD3 (b), and QD4 (c) nanoconjugate samples (Scale bar = 100 µm).
Figure 4S: Zoomed images of HEK 293T cells in control (a) and after 24 h of incubation in direct contact with QD1 (b), QD2 (c), QD3 (d), and QD4 (e) nanoconjugate samples (Scale bar = 20 µm).
Figure 5S: Zoomed images of U87-MG cell images in control (a) and after 24 h of incubation in direct contact with QD1 (b), QD2 (c), QD3 (d), and QD4 (e) nanoconjugate samples (Scale bar = 20 µm).
