# Supplementary material for: One-Pot Aqueous Synthesis of Fluorescent Ag-In-Zn-S Quantum Dot/Polymer Bioconjugates for Multiplex Optical Bioimaging of Glioblastoma Cells
Source: Contrast Media Mol Imaging. 2017 Nov 13;2017:3896107. doi: 10.1155/2017/3896107 (PMC5702976; doi:10.1155/2017/3896107)
Supplement: Supplementary file 1 — Figure 1S: Schematic representation of fluorescent nanoconjugates based on AIS and ZAIS quantum dots with CMC ligands for multiplexed bioimaging of malignant glioma cells. Figure 2S: HEK 293T cells images after 24 h of incubation with QD2 (a), QD3 (b), and QD4 (c) nanoconjugate samples (Scale bar = 100 µm). Figure 3S: U87-MG cells images after 24 h of incubation with QD2 (a), QD3 (b), and QD4 (c) nanoconjugate samples (Scale bar = 100 µm). Figure 4S: Zoomed images of HEK 293T cells in control (a) and after 24 h of incubation in direct contact with QD1 (b), QD2 (c), QD3 (d), and QD4 (e) nanoconjugate samples (Scale bar = 20 µm). Figure 5S: Zoomed images of U87-MG cell images in control (a) and after 24 h of incubation in direct contact with QD1 (b), QD2 (c), QD3 (d), and QD4 (e) nanoconjugate samples (Scale bar = 20 µm). [file 3896107.f1.docx]

**One-Pot Aqueous Synthesis of Fluorescent Ag-In-Zn-S Quantum Dot/Polymer Bioconjugates for Multiplex Optical Bioimaging of Glioblastoma Cells**

Alexandra A. P. Mansur,^1^ Herman S. Mansur,^1,^^[[1]](#footnote-1)^* Sandhra M. Carvalho,^1,2,3^Anderson J. Caires^1^

*^1^Center of Nanoscience, Nanotechnology and Innovation - CeNano^2^I, Federal University of Minas Gerais-UFMG, Brazil*

*^2^Department of Preventive Veterinary Medicine, Veterinary School, Federal University of Minas Gerais-UFMG, Brazil*

*^3^Department of Physiology and Biophysics, ICB, Federal University of Minas Gerais-UFMG, Brazil*

**corresponding author:* [*hmansur@demet.ufmg.br*](mailto:hmansur@demet.ufmg.br) *(HSM);*

**Supplementary Material**

**Graphical Abstract**

**LP 585**

**505/550**

**AIS + ZAIS**

**AIS + ZAIS**

**overlaid**

**AIS**

**ZAIS**

**CMC**

**Zn-S**

**Ag-In-S**

**AIS + ZAIS**

Figure 1S - Schematic representation of fluorescent nanoconjugates based on AIS and ZAIS quantum dots with CMC ligands for multiplexed bioimaging of malignant glioma cells.


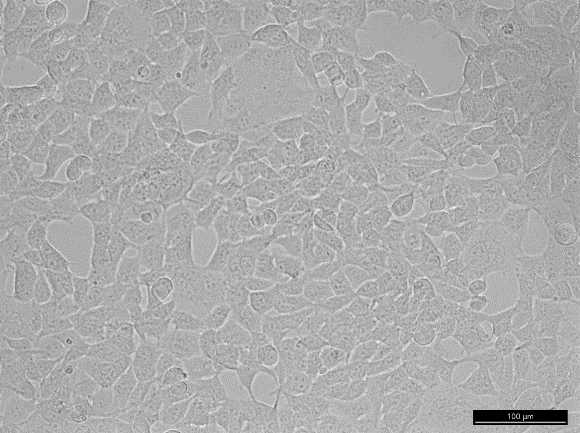

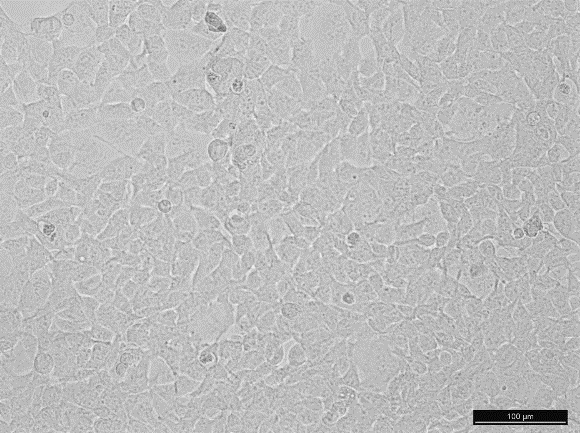


**(c)**

**(b)**

**(a)**


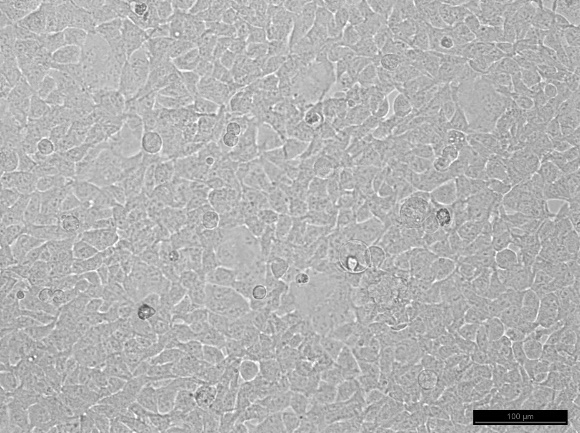


Figure 2S – HEK 293T cells images after 24 h of incubation with QD2 (a), QD3 (b), and QD4 (c) nanoconjugate samples (Scale bar = 100 µm).

**(a)**

**(b)**

**(c)**


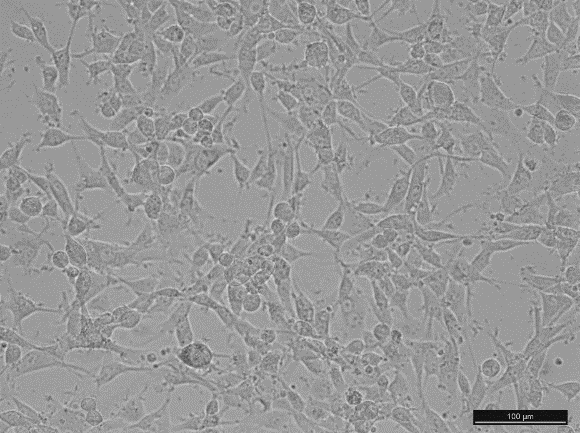

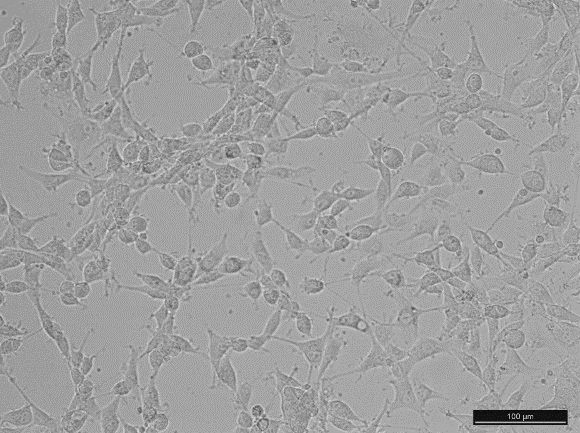


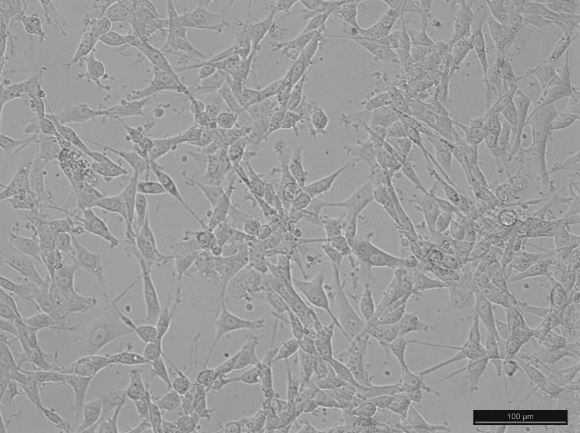


Figure 3S – U87-MG cells images after 24 h of incubation with QD2 (a), QD3 (b), and QD4 (c) nanoconjugate samples (Scale bar = 100 µm).


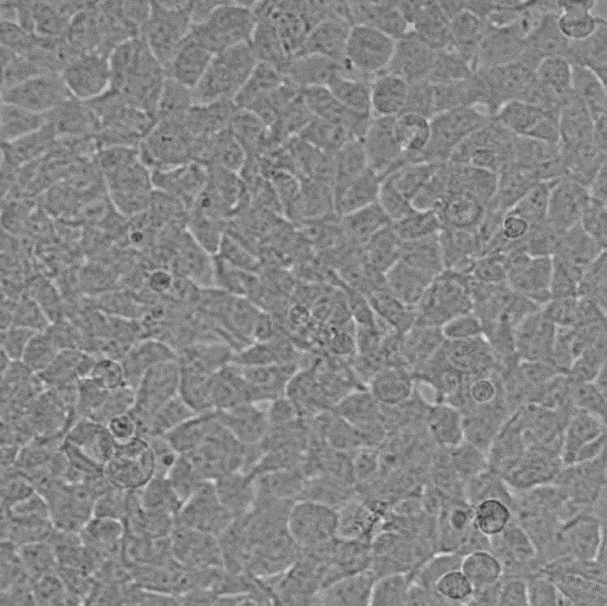

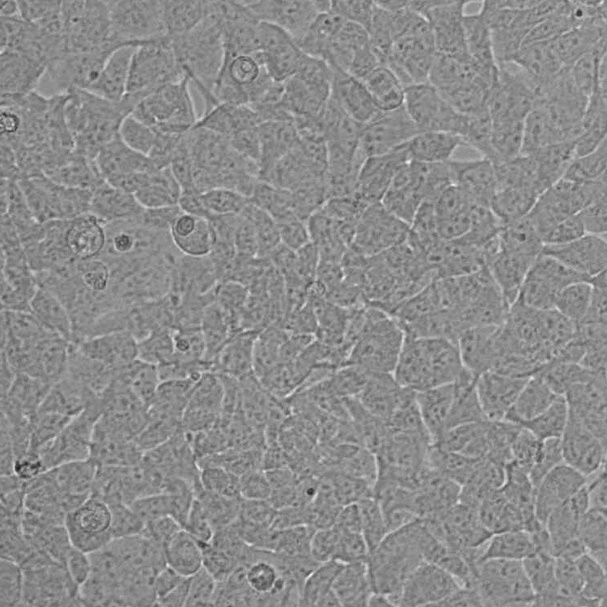


**(a)**

**(b)**

**(d)**

**(c)**


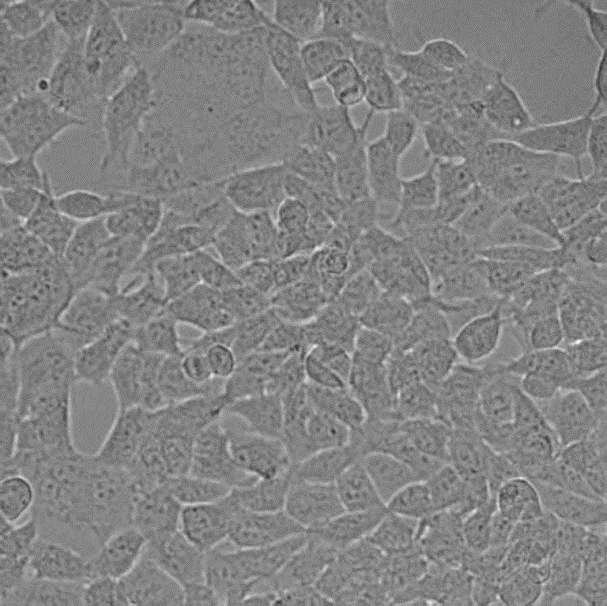

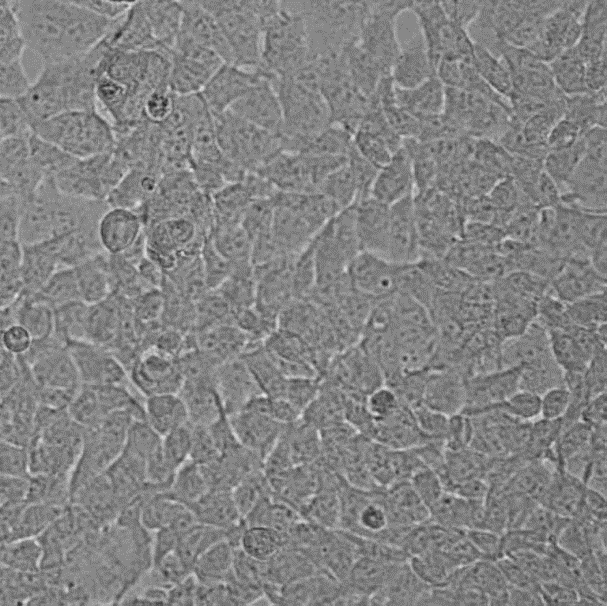


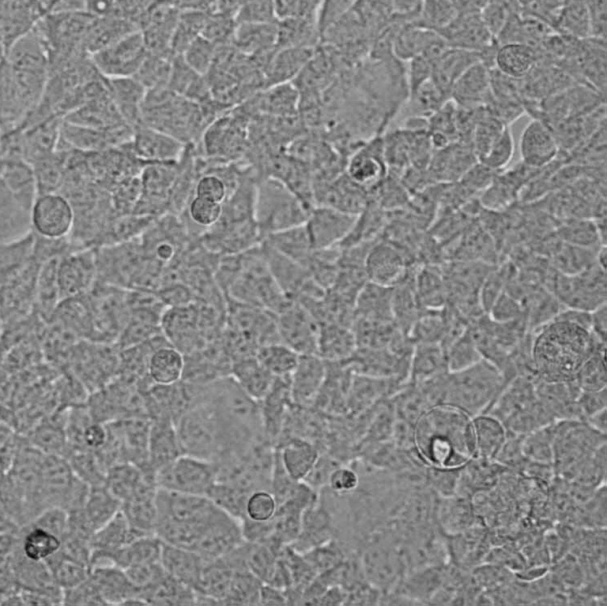


**(e)**

Figure 4S – Zoomed images of HEK 293T cells in control (a) and after 24 h of incubation in direct contact with QD1 (b), QD2 (c), QD3 (d), and QD4 (e) nanoconjugate samples (Scale bar = 20 µm).


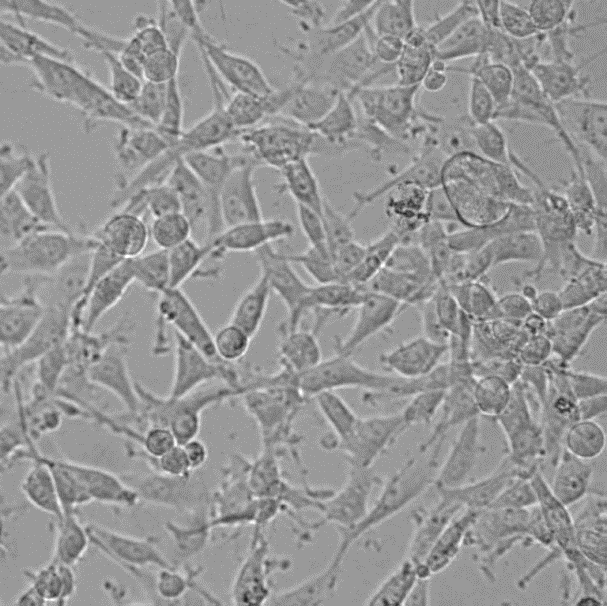

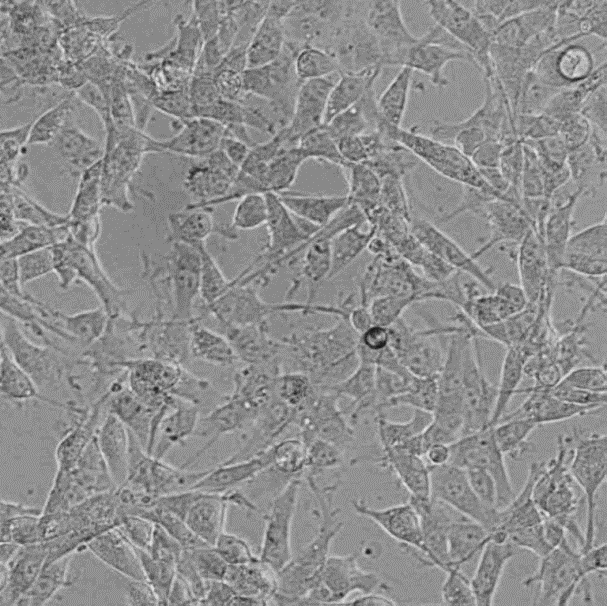


**(b)**

**(a)**

**(d)**

**(c)**


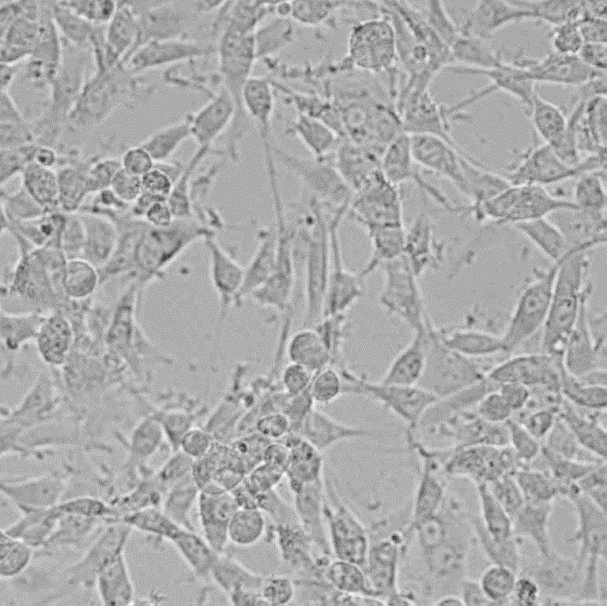

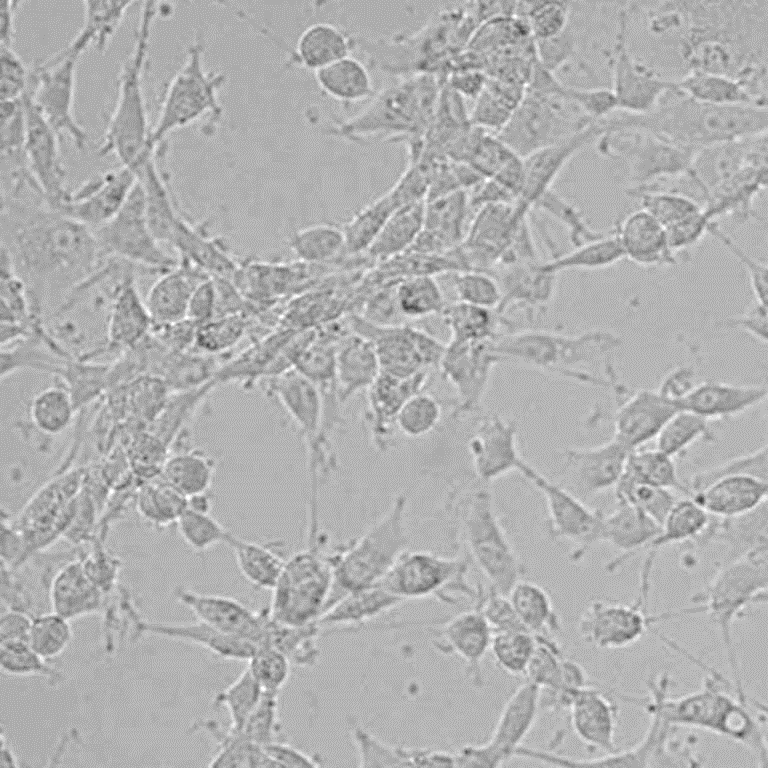


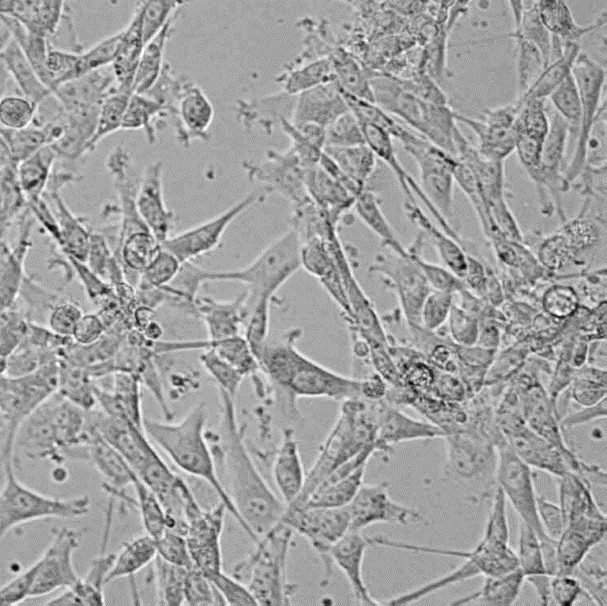


**(e)**

Figure 5S – Zoomed images of U87-MG cell images in control (a) and after 24 h of incubation in direct contact with QD1 (b), QD2 (c), QD3 (d), and QD4 (e) nanoconjugate samples (Scale bar = 20 µm).

1. * To whom correspondence should be addressed: Federal University of Minas Gerais, Av. Antônio Carlos, 6627 – Escola de Engenharia, Bloco 2 – Sala 2233, 31.270-901, Belo Horizonte/MG, Brazil; Tel: +55-31-34091843; Fax: +55-31-34091843; E-mail: [hmansur@demet.ufmg.br](mailto:hmansur@demet.ufmg.br) (H. Mansur) [↑](#footnote-ref-1)
